# Characteristics of Deaths Among Individuals in US Immigration and Customs Enforcement Detention Facilities, 2011-2018

**DOI:** 10.1001/jamanetworkopen.2021.16019

**Published:** 2021-07-07

**Authors:** Molly Grassini, Sophie Terp, Briah Fischer, Sameer Ahmed, Madeline Ross, Niels Frenzen, Elizabeth Burner, Parveen Parmar

**Affiliations:** 1 Department of Emergency Medicine, Keck School of Medicine of the University of Southern California, Los Angeles; 2 Gould School of Law of the University of Southern California, Los Angeles

## Abstract

**Importance:**

Concerns have been raised that substandard medical care has contributed to deaths in US Immigration and Customs Enforcement (ICE) detention facilities. After each in-custody death, ICE produces detainee death reviews, which describe the circumstances of the death and determine whether ICE Performance-Based National Detention Standards (PBNDS) were violated.

**Objective:**

To describe factors associated with deaths in ICE detention facilities.

**Design, Setting, and Participants:**

This case series used data extracted from detainee death reviews of deaths among individuals detained in ICE facilities for whom these reviews were available from January 2011 to December 2018.

**Exposures:**

All individuals were in the custody of ICE at the time of death.

**Main Outcomes and Measures:**

Data including demographic information, medical histories, recorded medical data, and reported violations of PBNDS were systematically extracted and summarized.

**Results:**

Among 71 individuals who died in an ICE detention facility during the study period, detainee death reviews were available for 55 (77.5%). Most were male (47 [85.5%]), and the mean (SD) age at death was 42.7 (11.5) years. Individuals resided in the US for a mean (SD) of 15.8 (13.2) years before detention and were in ICE custody for a median of 39 days (interquartile range, 9-76 days) before death. Most had low burdens of preexisting disease, with 18 (32.7%) having a Charlson Comorbidity Index score of 0 and 15 (27.3%) having a score of 1 or 2. A total of 47 deaths (85.5%) were attributed to medical causes and 8 (14.5%) to suicide. Markedly abnormal vital signs were documented in the death reviews before 29 of 47 deaths from medical causes (61.7%), and 21 of these 29 deaths (72.4%) were preceded by abnormal vital signs during 2 or more encounters with ICE personnel before death or terminal hospital transfer. Overall, 43 detainee death reviews (78.2%) identified PBNDS violations related to medical care, with a mean (SD) of 3.2 (3.0) deficiencies per detainee death review.

**Conclusions and Relevance:**

In this case series, deaths in ICE detention facilities from 2011 to 2018 occurred primarily among young men with low burdens of preexisting disease. Markedly abnormal vital signs preceded death or hospital transfer for most nonsuicide deaths. The PBNDS were violated in most detainee death reviews. These results suggest that additional oversight and external evaluation of practices related to medical and psychiatric care within ICE facilities are needed.

## Key Points

**Question:**

What were the characteristics of deaths among individuals detained in US Immigration and Customs Enforcement (ICE) facilities between 2011 and 2018?

**Findings:**

In this case series of 55 individuals who died in ICE detention facilities, most deaths occurred among young men with low rates of preexisting disease, with 14.5% of deaths attributed to suicide and 85.5% to medical causes. Death investigation records identified violations of ICE’s internal standards for delivery of health care in most of these deaths.

**Meaning:**

The findings suggest that additional oversight and external evaluation of practices related to medical and psychiatric care within ICE facilities are needed.

## Teaser

This case series described the characteristics of deaths among individuals detained in US Immigration and Customs Enforcement (ICE) facilities from 2011 to 2018.

## Introduction

Beginning in 2017, intensified efforts to deport immigrants increased the population in US Immigration and Customs Enforcement (ICE) detention facilities and the number of individuals requiring medical care from the ICE detention system.^1,2^ During fiscal year 2018 (October 1 to September 30, 2018), ICE detained 396 448 persons pending hearings on immigration claims, nearly half of whom would have previously been released on parole or with bond.^3,4^ As of September 10, 2019, 486 190 individuals had been detained during fiscal year 2019 (October 1 to September 30, 2019), resulting in a mean daily census of 49 403.^5^ Detained individuals include asylum seekers, individuals who overstay visas or otherwise remain in the US without legal documentation, and immigrants with certain types of criminal convictions and may include those with undocumented immigration status who have come in contact with the criminal justice system.

Between 2003 and 2017, 172 people died while detained in ICE facilities.^6^ A 2019 report by the US Department of Homeland Security Office of the Inspector General highlighted concerns regarding conditions faced by individuals in ICE detention facilities.^7^ However, information on the circumstances of deaths in ICE detention facilities is currently limited. The ICE Office of Detention Oversight initiates investigations resulting in detainee death reviews (DDRs) after each death that occurs in ICE detention facilities. These investigations involve interviews of detention facility staff, individuals in detention, medical record review, and review of security footage and logs. The DDRs are most often conducted by private contractors.^8,9^ These reviews generally identify whether Performance-Based National Detention Standards (PBNDS) were appropriately followed. Adapted from 2000 National Detention Standards (NDS), PBNDS were implemented in 2008 and revised in 2011 and 2016; they were revised once more and renamed the NDS in 2019.^10^ The NDS/PBNDS (hereafter referred to as PBNDS) prescribe standards of safety, security, and care for ICE detention facilities.^10^

Reports on a limited selection of DDRs written by Human Rights Watch^11^ and the American Civil Liberties Union (ACLU)^12^ highlighted widespread health systems issues across multiple facilities contributing to deaths among individuals detained by ICE. Human Rights Watch evaluated 15 DDRs and identified a number of dangerous inadequacies, including practitioners failing to interpret basic medical data and appropriately treat acute conditions, problematic use of solitary confinement (also known as segregation) for people with psychosocial disabilities, and flawed emergency responses.^11,13^ The ACLU evaluated 8 DDRs and uncovered similar failures.^12^ Evidence of noncompliance with ICE’s own medical standards was found in all of the DDRs reviewed by both the ACLU and Human Rights Watch.^11,12^ These findings are consistent with a report published by the Department of Homeland Security Office of the Inspector General after unannounced visits to 4 ICE detention facilities in 2019.^7^ The Office of the Inspector General identified PBNDS violations at all facilities visited during its review.^7^

Prior published reports^6,14^ highlighting deficiencies in medical and psychiatric care associated with deaths in ICE detention have been based on review of a relatively small number of DDRs and limited government inspections. However, to date, no systematic evaluation of a large subset of DDRs has been published in the peer-reviewed medical literature. We sought to describe systemic factors that may have been associated with deaths in ICE detention facilities between January 2011 and December 2018.

## Methods

This case series examined DDRs for deaths among individuals detained in ICE facilities between January 2011 and December 2018. The DDRs were obtained from an online repository or from civil rights organizations known by us to have litigated for access to these reports. These organizations were, in turn, asked to refer us to other individuals who might be able to provide additional DDRs. Among 71 reported deaths between 2008 and 2011 in ICE detention facilities, DDRs were available for 55. This study was deemed exempt by the University of Southern California institutional review board, with a waiver of informed consent because publicly available data were used. This study followed the reporting guideline for case series.^15^

Produced by the ICE Office of Professional Responsibility and Office of Detention Oversight, many DDRs are publicly accessible via the official Department of Homeland Security ICE web page.^16^ These reports, ranging in length from 12 to 188 pages, include a brief synopsis of the individual’s immigration and medical history, a description of the detention facility, and a narrative summary of the events leading up to the death. This narrative summary is developed from a combination of medical record review and interviews with medical and security staff as well as other detained individuals. A review of video footage and security logs is conducted when these resources are available. Each report concludes with a list of PBNDS violations identified in the review and, at the DDR writer’s discretion, descriptions of additional security or medical care concerns noted by the reviewer.

Two of 3 independent researchers (M.G., B.F., P.P.) extracted data from each DDR using a data extraction form developed in REDCap.^17^ The senior researcher (P.P.) reviewed data extractions and highlighted all differences between the 2 independently completed data extraction forms. All discrepancies were resolved by consensus among all 3 data extractors after another collaborative review of the relevant DDR. This consensus review was conducted on a weekly basis. Any discrepancies on which the 3 data extractors could not agree were resolved by consensus of the most qualified research team members (for medical questions, S.T. and E.B.; for legal questions, N.F.).

Data extracted included descriptions of deceased individuals’ demographic information and immigration history and the chronology of ICE detention, including dates of detention, transfers, and death. In addition, characterizations of detention facility type (privately owned, ICE operated, or contracted by an intergovernmental service agreement whereby ICE pays for beds in local prisons and jails) and location were extracted.^3,18^ We extracted data from each DDR regarding the location where the individual died (ICE detention facility, in transport to the hospital, emergency department, or other hospital ward), the method used to transport the individual to a higher level of care when applicable, whether cardiopulmonary resuscitation (CPR) was initiated and under what circumstances (to describe the characteristics of the emergency response in ICE facilities), information about the individual’s medical and psychiatric history, whether past medical information had not been disclosed by the individual who died, and listed cause of death. We also recorded instances in which medical or security staff or other detained individuals had raised concerns about the deceased individual’s health to a supervisor or other staff member in the days preceding death. We noted whether the deceased individual had themselves filed a grievance related to their health before their death. Deaths were categorized as caused by suicide (if suicide was listed as a cause of or contributor to death) or medical (if suicide was not included as a cause of death). For deaths attributable to suicide, we noted whether the individual was prescribed and taking psychiatric medications at the time of death and whether the individual was under observation for suicide risk at the time of death or at any point earlier during detention.

Medical deaths were categorized as being attributable to infectious diseases, noncommunicable diseases, or both based on documented causes of death. Reports of death from any medical cause were also reviewed for severely abnormal vital signs before death using criteria established in prior work.^19^ Multiple severely abnormal vital signs measured simultaneously were counted as 1 episode (eg, tachycardia and hypotension noted during vital sign reading for an individual with sepsis would qualify as 1 episode of abnormal vital signs). We noted comorbidities and used these to calculate each individual’s Charlson Comorbidity Index (CCI) score.^20,21^ The CCI is a validated tool that considers age and preexisting medical conditions to predict 10-year mortality for an individual; scores range from 0 to 24, with higher scores reflecting increased burden of disease.^20,21^ Comorbidities were primarily extracted from the DDRs. We also included comorbidities cited as preexisting during hospitalizations and at autopsy to account for underdiagnosis of chronic conditions in this population, which was likely to have had limited access to primary care before detention owing to legal status. In addition, if multiple elevated blood pressures were measured during clinical evaluations while the individual who died was detained, these readings were reviewed by us to determine if the individual had grade 1 or higher hypertension using American Heart Association criteria.^22^

Furthermore, DDRs were reviewed to assess which PBNDS were specifically identified as having been violated in the DDRs. The specific version of PBNDS varied between reports, depending on the date of death and which standard (ie, 2008, 2011, or 2016) was in effect at each facility on the date of death. Given that most cases reviewed (n = 42) were evaluated under the 2011 PBNDS, deficiencies noted in death summaries referencing alternate PBNDS editions were normed on the 2011 PBNDS. These included 4 DDRs under NDS 2000, 8 under PBNDS 2008, and 1 under PBNDS 2016. Violation wording was collated among the various review versions and aligned with the 2011 PBNDS to standardize reporting of violations across the entire study population. Deficiencies in PBNDS standards were tallied for each DDR and stratified by respective violation subcategories.

### Statistical Analysis

Individuals’ characteristics, medical care circumstances, and PBNDS violations were analyzed descriptively, with data given as means and 95% CIs or medians and interquartile ranges as appropriate. We also describe 2 case studies (eAppendices 1 and 2 in the Supplement) to provide contextualized examples of factors that preceded death. Data analysis was performed using Stata, version 15.1 (StataCorp LLC).

## Results

Among 71 individuals who died while in an ICE detention facility during the study period, DDRs were available for 55 (77.5%). Demographic and immigration characteristics of these 55 individuals are summarized in Table 1. Among those who died, 47 (85.5%) were male; the mean (SD) age at death was 42.7 (11.5) years. Individuals who died had lived in the US for a mean (SD) duration of 15.8 (13.2) years before entering an ICE detention facility and spent a median of 39 days (interquartile range, 9-76 days) in ICE custody before death. There were 24 different citizenships represented among the 55 individuals who died. Thirty-four of the 55 deaths (61.8%) occurred in privately owned, for-profit detention facilities.

**Table 1.** Demographic and Immigration History of the 55 Individuals Who Died in ICE Detention Facilities and Had DDRs Available From 2011 to 2018

Characteristic	Individuals (n = 55)
Age at death, y	
Mean (SD)	42.7 (11.5)
Median (IQR)	45 (33-53)
Documented entries into the US, No.	
Mean (SD)	1.6 (1.2)
Median (IQR)	1 (1-2)
Time in the US before ICE detention, y	
Mean (SD)	15.8 (13.2)
Median (IQR)	15 (4-22)
Time in custody before death, d	
Mean (SD)	120.6 (288.7)
Median (IQR)	39 (9-76)
Gender, No. (%)	
Male	47 (85.5)
Female	7 (12.7)
Transgender (male to female)	1 (1.8)
Citizenship, No. (%)	
Mexico	11 (20)
Honduras	8 (14.6)
Guatemala	8 (14.6)
El Salvador	6 (10.9)
Cuba	3 (5.5)
Other^a^	19 (34.6)
Criminal detention immediately preceding ICE detention, No. (%)	
Yes	25 (45.5)
No	30 (54.5)
State of criminal detention, No. (%)	
Texas	6 (24.0)
California	4 (16.0)
Florida	3 (12.0)
Other	12 (48.0)

Abbreviations: DDR, detainee death review; ICE, Immigration and Customs Enforcement; IQR, interquartile range.

^a^ Antigua and Barbuda (n = 1), Armenia (n = 1), Bosnia-Herzegovina (n = 1), Brazil (n = 1), Canada (n = 1), Dominican Republic (n = 1), Ecuador (n = 1), France (n = 1), Gabon (n = 1), India (n = 1), Iran (n = 1), Jamaica (n = 2), Laos (n = 1), Mozambique (n = 1), Nicaragua (n = 1), Nigeria (n = 1), Panama (n = 1), and Russia (n = 1).

Circumstances of deaths are given in Table 2. Individuals who died had a low burden of preexisting disease. Most had CCI scores of 0 (18 [32.7%]) or 1 to 2 (15 [27.3%]), correlating with an expected 10-year survival of 98% and 90% to 96%, respectively. In 2 instances, the person who died had known medical conditions that they did not initially report to ICE medical staff (diabetes [n = 1], HIV infection [n = 1]). In addition, substance withdrawal syndromes contributed to 2 deaths (methadone [n = 1], alcohol [n = 1]).

**Table 2.** Circumstances of Death Among Persons Who Died in ICE Detention Facilities

Characteristic	Deaths, No./total No. (%)
Location of death	
Detention facility	4/55 (7.3)
Hospital	
Emergency department	16/55 (29.1)
Inpatient	35/55 (63.6)
Among those transported, method of transport	
Detention facility vehicle	14/51 (27.5)
Emergency medical service	36/51 (70.6)
Unable to determine	1/51 (2.0)
CPR initiated	
Yes	42/55 (76.4)
No	13/55 (23.6)
CPR initiator	
Other detained person	2/42 (4.8)
Detention facility staff	18/42 (42.9)
Emergency medical service personnel	4/42 (9.5)
Hospital inpatient staff	18/42 (42.9)
Concerns about condition were raised in days leading up to death or identification of critical illness	
Yes	15/55 (27.3)
No	40/55 (72.7)
Individuals who raised concerns	
Detention security staff	5/15 (33.3)
Detention medical staff	4/15 (26.7)
Other individuals in detention	6/15 (40.0)
Abnormal vital signs recorded in detention^a^	
Yes	29/55 (52.7)
No	26/55 (47.3)
Charlson Comorbidity Index score	
0	18/55 (32.7)
1-2	15/55 (27.3)
3-4	14/55 (25.5)
≥5	8/55 (14.5)
Cause of death	
Medical	47/55 (85.5)
Noncommunicable disease	29/47 (61.7)
Infectious diseases	10/47 (21.3)
Both noncommunicable and infectious diseases	8/47 (17.0)
Suicide and self-harm	
All	8/55 (14.5)
Hanging	6/8 (75.0)
Other asphyxiation	1/8 (12.5)
Overdose	1/8 (12.5)
Received suicide precautions or under observation for suicide risk at the time of death	0/8
Under observation for suicide risk at any time during detention	4/8 (50.0)
Prescribed psychiatric medications at the time of death	6/8 (75.0)
Taking psychiatric medications at the time of death	6/8 (75.0)
Known medical condition not reported to ICE	2/55 (3.6)
Death associated with substance withdrawal	2/55 (3.6)
Receiving comfort care at time of death	2/55 (3.6)

Abbreviations: CPR, cardiopulmonary resuscitation; ICE, Immigration and Customs Enforcement.

^a^ Abnormal vital signs were defined as a heart rate greater than 99 beats per minute or less than 60 beats per minute, a systolic blood pressure greater than 179 mm Hg or less than 90 mm Hg or a diastolic value greater than 109 mm Hg, a respiratory rate greater than 24 breaths per minute or less than 12 breaths per minute, a temperature below 35.0 °C (95 °F) or above 37.9 °C (100.2 °F), or an oxygen saturation of less than 90%.^19^

Of 55 deaths, 47 (85.5%) were attributed to medical conditions. Markedly abnormal vital signs were documented in the death reviews before 29 of 47 deaths from medical causes (61.7%), and 21 of these 29 deaths (72.4%) were preceded by abnormal vital signs during 2 or more encounters with ICE personnel before death or terminal hospital transfer. Twenty-nine of the 47 medical deaths (61.7%) were attributed to noncommunicable diseases (eg, cancer, stroke), and 10 (21.2%) to communicable diseases (eg, tuberculosis, other contagious infections). Eight individuals (17.0%) died of a combination of both communicable and noncommunicable diseases.

Six reports (10.9%) noted that a fellow detained person had raised concerns about the physical or mental health of the person who died before their death. Nine reports (16.4%) noted that similar concerns had been raised by detention facility security or medical staff. Forty-two of the 55 individuals who died (76.4%) received CPR before death. In 18 instances, CPR was initiated by detention facility staff, and in 2 instances, CPR was started by another individual in ICE custody. In 4 instances, emergency medical service personnel initiated CPR, twice on arrival to the ICE facility in the presence of ICE medical staff responders and twice during transport. Overall, 20 individuals (36.4%) died before hospital admission, 4 in the detention facility and 16 in the emergency department within an hour of arrival.

Characteristics of deaths attributed to suicide are included in Table 2. Among the 8 deaths attributed to suicide, 6 were by hanging, 1 by asphyxiation after an apparent intentional ingestion of a foreign object (a sock containing a whole toothbrush causing airway blockage), and 1 by an apparent intentional overdose of prescribed tricyclic antidepressants that the individual had been stockpiling before death. None of these individuals were under observation for suicide risk at the time of their death, although 4 had been at some point earlier during their detention.

A complete list of PBNDS categories and a summary of DDRs that identified PBNDS violations by category are included in Table 3. Of 55 DDRs, 43 (78.2%) identified PBNDS violations related to care, a category of standards that includes subcategories such as hunger strikes; medical care; significant self-harm and suicide prevention and intervention; and disability identification, assessment, and accommodation.^23^ Characteristics of PBNDS violations in the care category are summarized in Table 4. All DDRs with PBNDS violations in the category of care involved deficiencies in medical care specifically, with a mean (SD) of 3.2 (3.0) deficiencies involving medical care per case. In addition, within the care category, 5 of 43 DDRs (11.6%) identified concurrent deficiencies in the subcategory of significant self-harm and suicide prevention and intervention.

**Table 3.** Total DDRs With PBNDS Violations Identified by Category

PBNDS category	DDRs with a violation, No. (%) (n = 55)	Cited violations, No.
Safety	12 (21.8)	17
Security	27 (49.1)	85
Order	1 (1.8)	3
Care	43 (78.2)	191
Activities	1 (1.8)	1
Justice	1 (1.8)	1
Administration and management	1 (1.8)	1

Abbreviations: DDRs, detainee death reviews; PBNDS, Performance-Based National Detention Standards.

**Table 4.** Performance-Based National Detention Standards Violations for Care Standards by Subcategory

Subcategory	DDRs with violations, No. (%)	Total violations, No.	Violations per DDR, mean (range), No.
Food service	1 (1.8)	1	0.02 (0.13)
Hunger strikes	1 (1.8)	1	0.02 (0.13)
Medical care	43 (78.2)	174	3.16 (2.95)
Medical care (women)	2 (3.6)	2	0.36 (0.27)
Personal hygiene	1 (1.8)	1	0.02 (0.13)
Significant self-harm and suicide prevention and intervention	5 (9.1)	10	0.20 (0.61)
Terminal illness, advance directives, and death	2 (3.6)	2	0.02 (0.19)
Disability identification, assessment, and accommodation	0	0	0 (0)

Abbreviation: DDRs, detainee death reviews.

Case studies and PBNDS violations of 2 deaths of individuals in ICE custody that were preceded by multiple documented measurements of abnormal vital signs are given in eAppendices 1 and 2 in the Supplement.

## Discussion

In this case series, we describe 55 deaths that occurred in ICE detention facilities between 2011 and 2018. Most deaths were attributed to medical causes, primarily noncommunicable diseases. Delayed or inappropriate care, lack of response to markedly abnormal vital signs, and slow or lack of initiation of CPR were described in the DDRs obtained. We also identified PBNDS violations in the most the DDRs evaluated. A total of 78.2% of DDRs cited violations of ICE’s internal medical care standards, suggesting repeated and systemic failures across multiple areas of health care. These violations of PBNDS generally were associated with individuals not receiving timely and appropriate medical care. Violations occurred in multiple facilities nationwide and, in most cases, were directly associated with the deaths of the individuals. These findings are consistent with trends highlighted by Human Rights Watch, the ACLU, and the Office of the Inspector General.^7,12,13^

Signs and symptoms suggestive of critical medical illnesses were missed in the days preceding many of the described deaths. A total of 52.7% of these individuals had severely abnormal vital signs during at least 1 medical evaluation at the detention facility before their death, including 1 individual who had 22 episodes of abnormal vital signs documented and another who had 12. In the 2 case studies described in eAppendices 1 and 2 in the Supplement, vital sign abnormalities were minimized or dismissed by the ICE medical care team. In multiple cases, detention facility staff or other individuals in the detention facility raised concerns about an individual’s health to a superior or staff member before the individual’s death; it is possible that these deaths might have been avoided if these concerns had been addressed. Although CPR was appropriately initiated in many cases, cases in which CPR was initiated by individuals other than ICE medical staff provide insight into potential improvements in ICE facility emergency responses.

Warning signs for critical psychiatric illnesses were also missed. Of 8 individuals with death attributed to suicide, 4 had been under observation for suicide risk at some point during detention, but none of these individuals were under observation for suicide risk at the time of death. Violations of standards of psychiatric care occurred in many of these deaths. Improved psychiatric treatment and monitoring in ICE detention facilities may be required to address these findings.

Of note, the mean age at death among individuals who died in ICE detention facilities was 42.7 years, which is substantially younger than the typical life expectancy for individuals not born in the US (81.2 years for men, 85.1 years for women).^24^ In addition, these individuals spent a median of 39 days in ICE custody before their death. The duration of detention preceding death suggests that individuals in ICE detention do not die due to injuries sustained or diseases contracted during border crossing efforts immediately before their detention or after spending many years in facilities while awaiting trial or deportation. Instead, these findings suggest that failures with regard to delivery of health care to those in ICE detention facilities were associated with premature deaths in a substantial proportion of the cases reviewed.

Deaths in ICE detention increased substantially in 2020, and there have been concerns raised about substandard care delivery between 2017 and 2020, particularly in the context of the current COVID-19 pandemic.^25,26,27,28^ Many individuals died within days of being released from ICE detention facilities, and many deaths never underwent the mandatory death review process, potentially leading to an undercount of mortality in ICE detention facilities nationwide.^29^ Department of Homeland Security officials should ensure timely, public, transparent reporting and independent medical reviews of all deaths in ICE detention facilities, including those that occur within 1 week of release. Facilities with recurrent violations should undergo targeted rehabilitation and close monitoring. Those that are unable to meet predetermined benchmarks and fail to implement acceptable corrective action should be faced with penalties including possible closure. These processes are vital to ensuring that the dignity and health of the detained population are respected.

### Limitations

This study has limitations. The DDRs typically contained multiple redactions and generally supplied limited or no medical records. Only 55 DDRs were available for 71 deaths, representing potential sampling bias because the circumstances of the other cases were not available for review. Medical information was often summarized in the review, and physical copies of original medical records containing these data were not consistently included. The CCI was used to characterize the comorbid conditions of the individuals who died in this data set. The CCI is typically used for preprocedural risk assessment, and only conditions that were mentioned by the individual in detention or noted on medical review were considered, not conditions that may have been ignored or unidentified by personnel in the facility. We corrected for this by including retrospective diagnoses of all medical conditions; however, our additions may have overestimated disease burden and elevated the CCI scores. Furthermore, we were unable to obtain medical records for a comparison group. Medical records of individuals with similar medical comorbidities and demographic characteristics who did not die while in ICE detention or those who were released into communities rather than being placed in detention could have been potential comparators but were not available to us owing to privacy considerations. Future studies might be conducted in collaboration with ICE, ideally to follow cohorts of individuals released into the community and those kept in detention facilities, for example, to identify differences in health outcomes prospectively.

## Conclusions

In this case series, deaths in ICE detention facilities from 2011 to 2018 occurred primarily among young men with low burdens of preexisting disease. Markedly abnormal vital signs preceded death or hospital transfer for most nonsuicide deaths. The PBNDS were violated in most DDRs. These results suggest that additional oversight and external evaluation of practices related to medical and psychiatric care within ICE facilities are needed.

## Notes

Supplement. **eAppendix 1.** Illustrative Case Study 1 **eAppendix 2.** Illustrative Case Study 2
