# Supplementary material for: Characteristics of Deaths Among Individuals in US Immigration and Customs Enforcement Detention Facilities, 2011-2018
Source: JAMA Netw Open. 2021 Jul 7;4(7):e2116019. doi: 10.1001/jamanetworkopen.2021.16019 (PMC8264644; doi:10.1001/jamanetworkopen.2021.16019)
Supplement: Supplement. — eAppendix 1. Illustrative Case Study 1 eAppendix 2. Illustrative Case Study 2 [file jamanetwopen-e2116019-s001.pdf]

Supplemental Online Content

Grassini M, Terp S, Fischer B, et al. Characteristics of deaths among individuals in US Immigration and Customs Enforcement detention facilities, 2011-2018. *JAMA Netw Open.* 2021;4(7):e2116019. doi:10.1001/jamanetworkopen.2021.16019

eAppendix 1. Illustrative Case Study 1

eAppendix 2. Illustrative Case Study 2

This supplemental material has been provided by the authors to give readers additional information about their work.

© 2021 Grassini M et al. *JAMA Network Open.*

**eAppendix 1. Illustrative Case Study 1**
A 51-year-old citizen of Mexico with a reported history of smoking was held at an ICE processing center in New Mexico. Five days after entering the facility, he submitted two sick call requests for "symptoms including fever, sore throat, sneezing, runny nose, and flu". He was evaluated the next day and prescribed an anti-pyretic and antihistamine, and given return precautions. Five days later he submitted another sick call request because he had not improved and was scheduled for next day care. However, approximately 12 hours later, he was noted to appear ill and sent to the facility medical clinic for evaluation.<br><br>In the facility medical clinic, his temperature was noted to be 104°F with a "moderately elevated" pulse, and "diminished" oxygen saturation (no numeric data was provided in the DDR reporting from this episode). The man was diagnosed with an "upper respiratory infection of bacterial origin" and prescribed albuterol nebulizer treatments to be administered as needed, as well as oxygen therapy consisting of oxygen at two liters by nasal cannula. Though the infection was deemed to be bacterial in nature, available records do not indicate whether antibiotics were ordered. Nursing staff were unable to administer physician-ordered albuterol due to a lack of equipment at the detention facility.<br><br>In the detention facility's Medical Housing Unit (MHU), a physician diagnosed this individual with chronic obstructive pulmonary disease (COPD) based on his history of smoking and existing symptoms of fever, congestion, and respiratory infection. Based on this diagnosis, the physician stated that as long as the individual's oxygen saturation remained above 85% without supplemental oxygen, he could be transferred out of the MHU to the general population.<br><br>During his time in MHU, multiple oxygen saturation readings of 80%, 82%, 83%, 84% were recorded, supplemental oxygen was administered intermittently, and at one point was increased from two to four liters nasal cannula without improvement in hypoxia. In total, there were 12 episodes of abnormal vital signs noted during the days leading up to this individual's death. On the day of transfer to a local hospital, nine days after the initial sick call, the medical record notes his oxygen saturation was 78%. A physician noted decreased breath sounds, and a chest x-ray showed an infiltrate in the left lung. He was admitted to the hospital and treated with antibiotics, bronchodilators, and additional supplemental oxygen. Three days later he was placed on bilevel positive airway pressure (BiPAP) and admitted to the intensive care unit where he was eventually intubated and died three weeks later of diffuse alveolar damage due to bronchopneumonia.<br><br>Upon review, the ICE Office of Detention Oversight (ODO) found OCPC to have been deficient with compliance with ICE PBNDS 2011 in six areas including three areas in the category of *Medical Care.* Citing ICE PBNDS 2011, *Medical Care, Section (V)(B), Designation of Authority*, which requires personnel to perform duties within their scope of practice for which they are credentialed by training licensure, certification, job description and/or written standing or direct orders by personnel authorized to give such orders. There was no documented provider order to raise the individual's oxygen therapy from two to four liters

© 2021 Grassini M et al. *JAMA Network Open.*

on specific dates. In reference to ICE PBNDS, *Medical Care, Section (V)(X)(9), Informed Consent and Involuntary Treatment* which requires medical staff to explain medical risks if care is declined and documentation of refusal and request for detained individuals to sign a refusal, staff failed to obtain written consent when the individual refused vital signs at a sick call request. Citing ICE PBNDS 2011, *Medical Care, Section (V)(S)(4), Delivery of Medication* which requires medications and medically necessary treatments to be provided on schedule, the individual was not administered prescribed nebulizer treatments due to lack of available tubing and mask.<br><br>The ODO also noted several additional concerns including failure of facility staff to document oxygen saturations in four instances, failure of RNs to notify a physician of low oxygen saturation, including multiple consecutive readings below the threshold set for possible return to the general population, and failure to monitor oxygen saturation following administration of supplemental oxygen.
Note: This exhibit provides a summary of findings reported in the DDR released by ICE

© 2021 Grassini M et al. *JAMA Network Open*.

**eAppendix 2. Illustrative Case Study 2**
A 36-year-old citizen of Guatemala with a reported history of head trauma and smoking, who was later found to also have cirrhosis upon autopsy, was initially held at a detention facility in Wisconsin. During his time at the first facility, the man sought medical care for blood in his stool, which was attributed to suspected internal hemorrhoids and for which he was prescribed a stool softener. The individual was transferred to a staging facility with a plan for his removal from the United States to Guatemala eight days later. After a screening chest X-ray was found to be concerning for tuberculosis, he was transferred from an ICE detention facility to a local hospital, where he was admitted for 22 days. During this time, he was diagnosed with tuberculosis. He underwent chest tube placement and was in the intensive care unit for several days pending chest tube removal. He was discharged back to the detention facility on rifampin, isoniazid, pyrazinamide, and ethambutol therapy for presumed pulmonary tuberculosis.<br><br>During his time at this facility, this individual was evaluated by outside medical providers on five separate occasions, including four visits to local hospitals; on at least one occasion, airborne precautions were reportedly not implemented during transfers. During his stay at the Louisiana detention facility, medical staff repeatedly engaged the man and took medical history in English without the use of an interpreter, though his primary language was noted to be Spanish. In total, 22 instances of abnormal vital signs were documented over the course of 36 days prior to the final hospital transfer preceding death**.** During this same period of time, he was advised by ICE facility medical providers to “increase his fluid intake” and to “change positions slowly” in order to address symptoms. Over the eight days preceding his terminal hospital transfer, this individual was noted to have abnormal vital signs on a daily basis, including temperatures of 101°F and 103°F, thirteen instances of pulses over 100 bpm and as high as 155 bpm, and blood pressure readings of 94/50 mmHg, 94/59 mmHg and 98/56 mmHg.<br><br>In the two days prior to the final hospital transfer, this individual began reporting shortness of breath, chest pain, and abdominal pain. On the date of final hospital transfer, he was noted to have a temperature of 103°F, a pulse of 134, respirations of 24, and a blood pressure of 86/54.[1] After transfer to a local hospital that sent him back to the detention facility after less than three hours, the individual was again noted to be hypotensive and was sent to another hospital where he was treated for sepsis, liver failure and transferred to the intensive care unit a large academic medical center one week later. Two weeks later, this individual died of an upper gastrointestinal hemorrhage due to cirrhosis with the contribution of emphysema and disseminated tuberculosis approximately three weeks after last transferred out of the detention facility.<br><br>Upon review, the ICE Office of Detention Oversight (ODO) found the detention facilities to have been deficient in their provision of medical care to this individual based on the ICE NDS 2000 and PBNDS 2011 standards, respectively. The ODO found the initial detention facility

© 2021 Grassini M et al. *JAMA Network Open.*

deficient in two standards related to training of detention center medical staff and the use of interpretation services during interactions with individuals with limited English language proficiency. The DDR cites ICE NDS 2000 *Medical Care Section (III)(D),* which states, "All new arrivals shall receive initial medical and mental health screening immediately upon their arrival by a health care provider or an officer trained to perform this function." Noting that the ODO could not locate any records or documentation that Officer [redacted] was trained in conducting intake screenings for detainees and that the Officer did not recall receiving any such training. The DDR also notes that while ICE NDS 2000 *Medical Care Section (III)(D)* states "If language difficulties prevent the health care provider/officer from sufficiently communicating…for the purposes of completing medical screening, the officer shall obtain translation assistance," finding that despite the individual's limited English language proficiency, nursing staff did not consistently use the telephonic interpretation service during encounters. Instead, they used other detainees as translators without knowledge of their proficiency or the detained individual's consent.<br><br>Additionally, the ODO found the second detention facility failed to comply with ICE PBNDS 2011 in nine areas, including two in the category of Medical Care. Noting ICE PBNDS 2011, *Medical Care, Section (V)(A)(2) Expected Practices* which requires facilities to directly or contractually provide detained individuals with medically necessary and appropriate medical, dental and mental health care and pharmaceutical services; the DDR cites numerous instances where vital signs were not documented on nursing rounds or measured abnormal vital signs were not reported or reasonably acted upon. With regards to ICE PBNDS 2011, *Medical Care, Section (V)(A)(8) Expected Practices* which requires facilities to provide its detainee population with language services necessary for detainees with limited English proficiency during any medical appointment, sick call treatment or consultation; the DDR noted that interpretation services were utilized by nurses only 20 percent of the time when communicating with the individual despite limited English proficiency.
Note: This exhibit provides a summary of findings reported in the DDR released by ICE

© 2021 Grassini M et al. *JAMA Network Open.*
